# Modifying the SERVPERF to assess paratransit minibus taxis *trotro* in Ghana and the relevance of mobility-as-a-service features to the service

**DOI:** 10.1016/j.heliyon.2021.e07071

**Published:** 2021-05-27

**Authors:** Emmanuel Dzisi, Daniel Atuah Obeng, Yaw Adubofour Tuffour

**Affiliations:** Regional Transport Research & Education Centre, Kumasi (TRECK), Department of Civil Engineering, Kwame Nkrumah University of Science and Technology, Kumasi, Ghana

**Keywords:** Paratransit, MaaS, Trotro, SERVQUAL, SERVPERF, Intelligent transportation systems

## Abstract

In Ghana, minibus taxis (*trotros*) are an important mode of transport that commute about 60% of the traveling public. In spite of their popularity, minibuses are generally inefficient, disorganized and have low service quality. In an attempt to assess service quality of the service, a modified SERVPERF tool was developed. Differences in perceptions of service quality between male and female respondents were also assessed, and the attractiveness of certain technological features as possible remedies to service quality issues were determined. Using an online Google forms version of the modified SERVPERF, responses from nearly one thousand commuters were collected. The link to the questionnaire was dispersed via social media (Whatsapp and Telegram) since the data was collected during the outbreak of COVID-19 in Ghana. Following a factor reduction, the most important service quality factors determined to affect *trotro* users were (i) Reliability of the service, (ii) Variability in cost and (iii) Responsiveness. Respondents also identified technologies that could help them (a) book, (b) report driver misbehavior, (c) make safe e-payments and (d) track the location of *trotros*, as most likely to improve their *trotro* service quality. The findings suggest that some mobility as a service features could have possible benefit for the trotro. The study is however limited in its ability to determine the exact impact of these technologies since it uses a stated preference approach. Future research could explore the willingness of other stakeholder groups such as operators in adopting these technologies since their participation would be key to the success of any such scheme.

## 1 Introduction

Paratransit in Ghana are the primary mode of transport for the majority of commuters. Since the mid-1960s however, the operations of the sector have seen little regulation, allowing for an influx of new entrants (Salifu, 2004), and an accompanying low service quality (World Bank, 2020). Hart notes that, post-colonially, the operators of this mode of transport have been viewed as exploitative and unreasonable in the pricing of their services. The combination of the low service quality and negative stereotypes that have endured over the years, have made the idea of improving the service seem daunting, if not impossible. This poor service quality and the failure of reform to address service challenges, are unfortunately borne by commuters, majority of whom have access to no other form of transportation.

Despite this, recent studies have argued for the introduction of smart technologies that can help improve the overall efficiency and service quality of the *trotro*. Adjaidoo and Akowuah (2021) as an example, used the dynamic rate leaky bucket algorithm to develop a departure scheduling model for these paratransit buses. This algorithm was aimed at improving the reliability of the service since the current service does not have any specific schedules for operation. Kommey et al. (2019) as well, developed “Trotro pass” a passenger accounting system that was designed to allow real time tracking of *trotros* while helping operators to estimate vehicle occupancy [and profitability]. The World Bank (2011) suggests that, technologies that can provide real time vehicle tracking and location, voice/data communication between vehicles and dispatchers, off-route monitoring, journey planning, route status information (for passengers via internet and mobile phone), fare collection, vehicle speed monitoring, etc. could significantly improve the service quality of the *trotro*. A review of literature, also suggests that some of these technological solutions, may well be needed in the trotro service (Hotor, 2016; Sam et al., 2018).

According to Vanderschuren and Baufeldt (2018), technology-based transport solutions could be one of the surest ways by which the inequalities of public transport can be addressed in developing countries. Schmidt (2013) also proposes that, for the paratransit, certain technological features can help improve user experience. Features that could allow commuters to; make advance arrangement for rides (passenger booking feature), e-payments to *trotro* drivers/mates (payment feature), cancel rides without a charge (ride-cancellation), track *trotros* in real time, report driver and mate misbehavior (reporting feature/trip evaluation feature), and use the digitized service with no-Wifi, were identified as being potential technology-based features of paratransit (Dzisi et al., 2021; Schmidt, 2013; Vanderschuren and Baufeldt, 2018).

Brakewood and Watkins (2019) as well identified that, technology-based transit solutions (such as real time transit information) made transit riders generally more satisfied with transit services. Technology-based transit solutions via cellphone [and other personal devices] also increased frequency of transit use (Brakewood et al., 2015a, Brakewood et al., 2015b; Ferris et al., 2010; Gooze et al., 2013; Tang and Thakuriah, 2012) and feelings of personal safety (Brakewood et al., 2014), while decreasing actual and perceived wait times of transit services (Brakewood et al., 2014; Brakewood et al., 2015a, Brakewood et al., 2015b; Ji et al., 2017; Watkins et al., 2011).

Based on these studies, it was determined that, the possibility of improving service quality of *trotros* using some technology-based solutions should be explored, particularly in light of the identified benefits. Technology-based solutions were preferred because they were assumed to have direct benefits for both paratransit operators and their users (Schmidt, 2013; The World Bank, 2011) and as well because they were considered low cost measures that could be implemented to effect significant change (Booysen et al., 2013).

### 1.1 Theoretical framework

In this study, it was assumed that the construct of service quality of *trotros*, was intrinsically linked to satisfaction. Satisfaction with trotro was also seen as being directly influential on commuters' desire to continue using *trotros*. van Lierop and El-Geneidy (2016) in a previous study, had determined that, transit users' satisfaction with the quality of service had positive effects on their satisfaction with the mode, which in turn, had a significantly positive effect on their loyalty/continued use. Cheng, Lai, Chen and Ou (2010), also determined that indeed, users' satisfaction was a key determinant in their willingness to repeatedly use mass transportation systems. These studies were however based on the fundamental marketing principle that customers’ satisfaction could greatly enhance repeat purchase intentions of the same and other products (Cardozo, 1965). This study therefore sought to determine service quality concerns of commuters, the possible disparities between service quality expectations of different commuter groups, and as well, the attractiveness of some technological solutions as potential remedies to service quality issues. In this study, it was hypothesized that understanding service quality issues of the *trotro* and possible factors that could discourage commuters (of all groups) from using it, could help in addressing the issues of this mode of transport. The technological solutions assessed were also hypothesized to create better satisfaction (Brakewood and Watkins, 2019) with *trotros* and have bearing on its continued use (Cardozo, 1965).

### 1.2 Objectives of the study

The primary objectives of this study, were:

(i) to modify the SERVPERF tool used in assessing the service quality concerns of *trotro* users (Agyemang, 2013; Sam et al., 2018)
(ii) to assess the differences in service quality as perceived by male and female respondents
(iii) to assess the attractiveness of certain technological features to commuters based on the service quality issues of *trotros* (Brakewood and Watkins, 2019; Schmidt, 2013; The World Bank, 2011).

### 1.3 SERVPERF in measuring transport service quality in Ghana

SERVPERF, the performance component of the Service Quality scale (SERVQUAL) was adopted in this study. The SERVPERF was preferred over the SERVQUAL in this study because it could reduce the number of questionnaire items by half (20 + perceptions items only), while achieving results that correlate well with SERVQUAL (Cronin and Taylor, 1994).

Together, both SERVQUAL and SERVPERF tools have been used extensively in research. In Ghana, the tools have been used in measuring service quality of the taxi service in Accra (Mensah and Ankomah, 2018), service quality of on-campus shuttle services (Ojo et al., 2014), and as well, the service quality of the bus transport services in Kumasi (Sam et al., 2018). In these studies, the five service quality constructs of Reliability, Empathy, Assurance, Tangibility and Responsiveness (Zeithaml et al., 1988) are measured. However, upon a closer look at the SERVQUAL constructs, one could realize that, strictly adopting these tools without recourse to the local transport service, would result in an incomplete or incorrect analysis of service quality experiences of commuters. This is because, the SERVQUAL (and the subsequent variation SERVPERF) which were developed initially as management tools for a formal, structured services industry (Cronin and Taylor, 1994; Parasuraman et al., 1991), would in such cases be used in measuring service quality for a service that is largely informal, and unstructured in operations. This study, in seeking to measure service quality issues of this informal transport service, sought to use constructs that were more relevant to the transport service under review. This was done because some constructs of the typical SERVPERF tool, were considered less relevant to trotros [based on literature and local experiences], while constructs that were considered more reflective of the service were not captured under a generic version of the tool (Birago et al., 2017; Dumedah, 2017; Dzisi et al., 2021; Mensah and Ankomah, 2018; Ojo et al., 2014; Poku-Boansi and Adarkwa, 2013; Sam et al., 2018; Tetteh et al., 2017).

In India, Verma, et al. (2017) used the SERVPERF in evaluating service quality of public transport following modifications. The modified tool was much more resemblant of the transit service operated in the city, and helped in the evaluation of challenges specific to women in Bangalore. In their study, Aniley & Negi (2010) also used a modified SERVPERF in determining passengers' overall satisfaction with HIGER buses in Ethiopia, while Pérez Sánchez et al. (2007) also used a traditional SERVPERF in examining the relationship between transport service quality and behavioral intentions in Spain. The findings from Pérez Sánchez et al. (2007) showed that, for the bus service, there indeed existed a relationship between the service quality and commuter's public transport use intentions. Sam et al. (2018) in a study of paratransit buses in Kumasi, Ghana, determined that only the construct ‘Reliability’ and ‘Responsiveness’ were significant in explaining service quality of *trotros*. This suggests that, for the paratransit service in Ghana, the factors important in explaining service quality may be different from the five dimensions used in explaining service quality elsewhere. For this reason, an exploratory study on these factors determining service quality was seen as appropriate, and modifications that made it possible to assess the service were implemented.

In this study, particular emphasis was placed on the construct ‘variability’ in service quality (Ndibatya et al., 2014). Variability occurs because the *trotro* service is informal and lacks formal structures of operation. This also leaves little room for addressing commuter complaints with the service (Abane, 2011). The informal nature of this service permeates almost all aspects of its operation including the fare structure, route selection, schedules (Ndibatya et al., 2014) and customer care (Abenaab, 2012; Ofori-Debra, 2017). This variability of service quality was therefore made a central theme of this study, just as it is a central theme of the operation of this mode. Variability in customer care as Ofori-Debra (2017) points out, can be observed in the differences in treatment meted out to persons living with disabilities for instance, or women (Anane, 2021) in comparison to able-bodied male commuters. The subsequent sections discuss the methodology of the study, the research questions, and the approaches used for the data collection and analyses.

## 2 Methodology

### 2.1 Study method

A 44-item version of the SERVQUAL tool was initially obtained. Following a review of literature, the three authors assessed the relevance of each of the items for their contextual applicability to *trotros*. The reviewers had professional and research backgrounds in public transport planning and management in Ghana, so their expertise was considered relevant in the selection of the items used in the survey. The final survey instrument after 3 rounds of reviews was set up online using Google forms, and the questionnaire publicized using social media platforms (Whatsapp and Telegram). The questionnaire was left online for a one-month period in the month of May 2020, and the link to the questionnaire was shared via these social media channels.

#### 2.1.1 Research questions

The following research questions were used as a guide in the study:

1. What are the important service quality issues affecting commuters?
2. What are the differences in service quality for male and female/PWD respondents?
3. What technology-based solutions would commuters find attractive as potential solutions to service quality challenges?

### 2.2 Primary data collection

For the data collection, purposive and convenience sampling approaches were used. The primary data used in this study was collected via the online survey. Using a six-point Likert scale with responses ranking from: 1 = strongly disagree, to 6 = strongly agree, respondents were expected to rate various service quality constructs. Respondents were as well encouraged to share the questionnaire with people within their social circles. The online survey was used because, at the time, it was considered the safest approach for the data collection, since the study was undertaken during the outbreak of the COVID-19 pandemic in Ghana.

Using the formula,

(1) n = N∗X / (X + N – 1),

where,

(2) $$\text{X}={{\text{Z}}_{\text{α}/2}}^{2}*\text{p∗}\left(1-\text{p}\right)/{\text{MOE}}^{2},$$

Z_α/2_ is the critical value of the Normal distribution at α/2.

MOE is the margin of error, p is the sample proportion, and N is the population size, an appropriate sample size was determined. The minimum sample size calculated based on a 95% confidence interval and a 4% margin of error was 601. The total number of responses received after the one-month period was 1,122. After data cleaning, the responses considered valid were 910, representing approximately 81% of responses received. Thompson (2004) points out that, for factor analysis, a sample size of N = 150 can be considered adequate if factors are defined by ten or more measured variables with structure coefficients <.40. Alternatively, a sample size of more than 300 can be considered adequate. Since the eventual sample used in this study met the requirement of 300 responses, the sample was considered adequate for the ensuing factor analysis.

The sample size was also, in comparison to previous studies, larger. Horsu and Yeboah (2015), Ayittah, et al. (2013) and Sam et al. (2018) for instance, used samples of 281, 200 and 103 in their respective studies on service quality. Additionally, in comparison to the country's population, the sample in this survey was considered fairly representative. About 97% of respondents fell within the 0–50 years age groups, and 42.2% of respondents fell between the 0–20 years age group. Comparatively, the national population had about 90% of people falling between the 0–54 age groups, and 56.08% between the age of 0–24 in 2020 (Indexmundi, 2020). The median age in the country was also 21.4 years (Indexmundi, 2020), as compared to the median age of 22.4 (median class of 20.5–30.5) of the respondents of this study.

### 2.3 Selection of participants

Research assistants were recruited to help circulate the questionnaire on social media. The research assistants were from various fields of endeavor (social, academic, religious, civil society, etc.) and were asked to share the link to the questionnaire across group channels and as well, directly to individuals. The research assistants also encouraged prospective respondents to share the questionnaire with other colleagues to ensure a large sample could be obtained.

### 2.4 Ethical consideration

Ethical clearance for the study was granted by KNUST. As part of the survey, respondents were informed that their responses would be analyzed as part of a larger study on service quality. Respondents were as well given assurances that their responses would be treated with anonymity (Joinson, 1999). Finally, respondents were informed about their right to refuse answering any question or the entire survey, if it made them uncomfortable.

### 2.5 Approach for analysis

#### 2.5.1 Factor analysis

According to Yong and Pearce (2013), factor analysis operates on the notion that measurable and observable variables can be reduced to fewer latent variables that share a common variance. Exploratory factor analysis in particular is used where there are possible latent relationships between various variables in a study. Under a factor analysis, the assumption is that, a factor

(3) *X*_j_ = a_*j*1_F_1_+ a_*j*2_F_2_+….. a_*jm*_F_*m*_ + e_*j*_

Where *p* = the number of variables (X_1_,X_2_,…,X_*p*_)
m = the number of underlying factors (F_1_, F_2_,...,F_m_)
*X*_j_ = the variables represented in latent factors

and

*j* = 1, 2… *p*

In this study, a Principal Component Analysis (PCA) was done, followed by a varimax rotation. A communality cut-off of 0.3 was also applied, ensuring only variables, with communalities above this threshold were included in the reduction. Naming of factors was also based on an evaluation of the central themes underlying the various factors.

#### 2.5.2 Comparison of service quality between groups

Since variability in service quality was a key service quality construct of interest, there was interest in examining if there were any significant differences in experiences of respondent groups. Female respondents and persons living with disabilities had been identified (Ofori-Debra, 2017; Sham et al., 2013; Verma et al., 2017) as some of the groups that experienced some forms of discrimination and poorer service quality, and as such, their responses to these service quality questions were compared with those of able-bodied male respondents. A parametric test in the form of an independent sample t-test was used afterwards in testing the significance of the differences in responses. This was aimed at providing an idea about the most prominent differences in perceptions of service quality based on the study parameters.

#### 2.5.3 Assessment of technology-based solutions

Besides identifying the service quality constructs affecting commuters, the attractiveness of technologies that were thought to improve upon some of the service quality issues of the *trotro* were assessed. Specifically, commuters were asked to select as many features as they thought could improve their travel experiences. The features presented to commuters included; a booking feature (that could allow commuters to make advance arrangement for rides), a payment feature (that could allow commuters to make e-payments to *trotro* drivers/mates using the app), a ride-cancellation feature (that could allow commuters to cancel rides without a charge), a real-time *trotro* tracking feature (that could help commuters readily locate the next available *trotro*), a driver and mate reporting feature (that could allow commuters to report driver/mate misbehavior), and a no-Wifi feature (that could allow commuters to use the MaaS service without WiFi). These features were selected based on (Brakewood and Watkins, 2019; Esztergár-kiss & Kerényi, 2019; Schmidt, 2013; The World Bank, 2011; Acheampong, 2021, Dzisi et al., 2020). Features that were observed to have more than 50% of the respondents select them were considered most in-demand for the *trotro* service, and most likely to highlight also, areas of concern as regards service quality.

## 3 Results

### 3.1 Descriptive statistics

From the data, majority (77.2%) of respondents stated they were regular users of the minibus *trotro* service. About thirty-nine percent (39%) of respondents were female, and 61% were male. Majority of respondents (83%) were below the age of 31 years and most (61%) stated they had an average monthly wage/income of GH¢ 500 (~$86) or less. Asked about their primary mode of transport, some 71% of respondents identified *Trotros* as their primary mode of transport. Taxis were the next most popular (10%), followed by walking (9%) and private vehicles (4%). Ride-hailing (4%) was the next most popular mode of transport, with bicycling constituting the least most popular mode of transport (2%).

It was also of interest to determine how long respondents had been using *trotros*. Majority of respondents (44%) stated they had used *trotros* for more than 11 years. Twenty-three percent (23%) stated they had used the *trotro* service between 6 to 10 years, nineteen percent (19%) stated they had used these minibuses between 2 to 5 years, and 14% stated they had used the service for less than a year.

In terms of the average number of trips they made a day, sixty six percent (66%) of respondents stated they made between 0 to 2 trips, 27% made between 3 and 4 trips a day, 5% made between 5 to 6 trips a day, and about 2% made 6 trips or more each day.

Respondents were also asked about their average daily spending on transport. About 12% stated they spent GH¢ 2 or less on transport, 29% stated they spent between GH¢ 3–4, 42% stated they spent between GH¢ 5–10, and 17% reported spending more than GH¢ 11 a day, on transport. Table 1 provides an overview of the characteristics of survey respondents and Table 2 shows a summary of the sampling adequacy and number of extracted factors.

**Table 1:** Characteristics of the survey participants.

Variable	N		(%)
Gender	Female	276	39.3%
	Male	427	60.7%
Age groups	a. ≤20	297	42.2%
	b. 21-30	287	40.8%
	c.31-40	71	10.1%
	d.41-50	27	3.8%
	e.51-60	19	2.7%
	f. above 60	2	0.3%
Monthly wage	a. ≤ GH¢500	431	61.3%
	b. GH¢ 501- GH¢1000	117	16.6%
	c. GH¢1001- GH¢2000	91	12.9%
	d.GH¢2001- GH¢5000	47	6.7%
	e.GH¢5001- GH¢10000	6	0.9%
	f. ≥ GH¢10001	11	1.6%
Primary mode of transport	a. Walking	63	9.0%
	b. Bicycling	12	1.7%
	c. Taxi	71	10.1%
	d. *Trotro*	502	71.4%
	e. Uber	24	3.4%
	f. Private vehicle	31	4.4%
How long have you been using *trotro*?	a. ≤1 year	96	13.7%
	b. 2–5 years	131	18.6%
	c. 6–10 years	166	23.6%
	d. ≥11 years	310	44.1%
No. trips a day	a. 0-2	465	66.1%
	b. 3-4	189	26.9%
	c. 5-6	32	4.6%
	d. >6	17	2.4%
Average daily spending on transport	a. ≤ GH¢ 2	88	12.5%
	b. GH¢ 3-4	202	28.7%
	c. GH¢ 5-10	292	41.5%
	d. ≥ GH¢ 11	121	17.2%
Intention to keep using trotro with improved service quality	Willing to use trotro	810	89%
	Unwilling to use trotro	100	11%

**Table 2:** Summary statistics on sampling adequacy and the number of extracted factors.

	Initial	Final
Items in the scale	22	19
Items deleted	0	3
Factors extracted	5	3
Total variance explained	56.98%	50.89%
KMO	87.0%	87.4%
Bartlett's test of sphericity	χ^2^ = 6455.34	Χ^2^ = 5826.01
Degree of freedom	231	171
∗p < 0.001		

### 3.2 Factor analysis

A factor analysis was then conducted to reduce the variables into fewer constructs. The Kaiser-Meyer Olkin (KMO) and Bartlett's test of sphericity were evaluated to determine the adequacy of the data for factor analysis. In this study, the KMO value obtained was approximately 0.874, which was significantly higher than the criteria defined by both (0.6 < KMO) (Kaiser, 1974) and (0.5 < KMO) (Hair et al., 1998), making it acceptable for the exploratory factor analysis. The Bartlett's test of sphericity (χ^2^: 5826.01, df: 171, Sig.: 0.000) also indicated that the variables for the factor analysis were significantly similar enough to each other, to undergo factor reduction. The factor reduction resulted in the extraction of three (3) factors which cumulatively, contributed 50.89% of the total variance. Respectively, the factors accounted for 24.9%, 14.6% and 11.4% of the variance. Factors with an Eigen value greater than 1 were extracted in accordance with the Guttman-Kaiser criterion (Guttman, 1954). The factors extracted were named *Reliability*, *Variability in cost*, and *Responsiveness*. Table 3 shows the rotated component matrix and the communalities of the variables in this study. The table also shows the variables that were deleted in the process of the factor analysis for having low communalities.

**Table 3:** Rotated component matrix.

	Component			Communalities
	1	2	3	
Rel1 Times between trotros are relatively short	0.570			0.336
Rel2 Passenger belongings are secure	0.606			0.404
Rel3 It is easy to get a space on a trotro in the morning	0.584			0.348
Rel4 It is easy to get a space on a trotro in the evening	0.562			0.319
Rel5 It is safe to use trotro in the morning	0.589			0.461
Rel6 It is safe to use trotro in the evening	0.592			0.399
Rel7 I have confidence in the trotro service operators to do their work	0.687			0.558
Rel8 Trotro drivers are polite	0.728			0.539
Rel9 Trotro mates are polite	0.758			0.597
Rel10 The insides of trotro buses are generally clean and hygienic	0.755			0.597
Rel11 Trotro mates are neat	0.712			0.593
VarC1 Trotro fares are sometimes higher for women, disabled		0.828		0.709
VarC2 Trotro fares are sometimes lower for women, disabled		0.734		0.619
VarC3 Women, disabled face more discrimination in using trotros		0.788		0.653
VarC4 Trotro fares vary for same the route during different times of day		0.557		0.336
Respon1 Trotro mates tend to be more helpful to disabled, women			0.795	0.639
Respon2 Trotro drivers tend to respond to passenger concerns			0.751	0.614
Respon3 Trotro fares are commensurate with service provided			0.681	0.558
Respon4 Fares charged for luggage are affordable			0.549	0.391
**Eigenvalues**	**24.899**	**14.631**	**11.368**	
Items deleted for cross loading and low communality			**Reason for deletion**	
VAR012 Most vehicles running as *trotros* are generally rickety			Low communality	
VAR013 *Trotro* drivers are often careless in their driving			Low communality	
VAR022 I only use *trotros* when I cannot afford other (better) modes of transport			Low communality	

### 3.3 Summary and reliability statistics of the SERVPERF scale

A basic descriptive and inter-item reliability test was done to assess the sub-scale of the 19-item SERVPERF scale (Table 4). The Cronbach's Alpha value of the identified factors were: Reliability (11 items), α = 0.866, Variability in cost (4 items), α = 0.785 and Responsiveness (4 items), α = 0.7088. The Cronbach alpha values determined also for each of these factors were greater than the minimum threshold of 0.70, suggesting there was reliability among the items of the final SERVPERF scale.

**Table 4:** Reliability statistics for the SERVPERF scale.

	Mean [SD]	Cronbach's Alpha	Alpha if item deleted
**Reliability**		**0.866**	
Rel1	3.18[1.55]		0.857
Rel2	3.07[1.55]		0.853
Rel3	3.14[1.71]		0.857
Rel4	2.83[1.58]		0.858
Rel5	3.68[1.60]		0.854
Rel6	3.09[1.55]		0.854
Rel7	3.25[1.52]		0.847
Rel8	2.95[1.39]		0.847
Rel9	2.59[1.39]		0.847
Rel10	2.55[1.43]		0.846
Rel11	2.26[1.39]		0.852
**Variability in cost**		**0.785**	
VarC1	2.61[1.799]		0.687
VarC2	2.69[1.81]		0.718
VarC3	2.91[1.82]		0.695
VarC4	3.55[1.79]		0.816
**Responsiveness**		**0.7088**	
Respon1	4.15[1.49]		0.655
Respon2	3.77[1.53]		0.606
Respon3	3.60[1.45]		0.613
Respon4	3.38[1.64]		0693

M: Mean; SD: Standard Deviation.

### 3.4 Comparison of service quality between groups

Following the independent sample t-test, statistically significant (**p<0.05**) differences were also observed between male and female respondents' responses to certain service quality parameters. The service quality parameters which drew statistically divergent responses were: ‘*Trotro* drivers are polite’, ‘*Trotro* mates are polite’, ‘*Trotro* mates are neat’ ‘*Trotro* *fares are sometimes higher for women, the old or disabled*’,‘*Trotro fares are sometimes lower for women, the old or disabled’, ‘Women, the* *old and disabled face more discrimination in using* *trotros*’, ‘*Trotro* *fares are sometimes higher for the same route during different times of day*’ and ‘I only use *trotros* when I cannot afford other (better) modes of transport’ . The results from this analysis are shown in Table 6 (see Appendix).

### 3.5 Attractiveness of technology-based solutions to commuters

**Figure 1:**
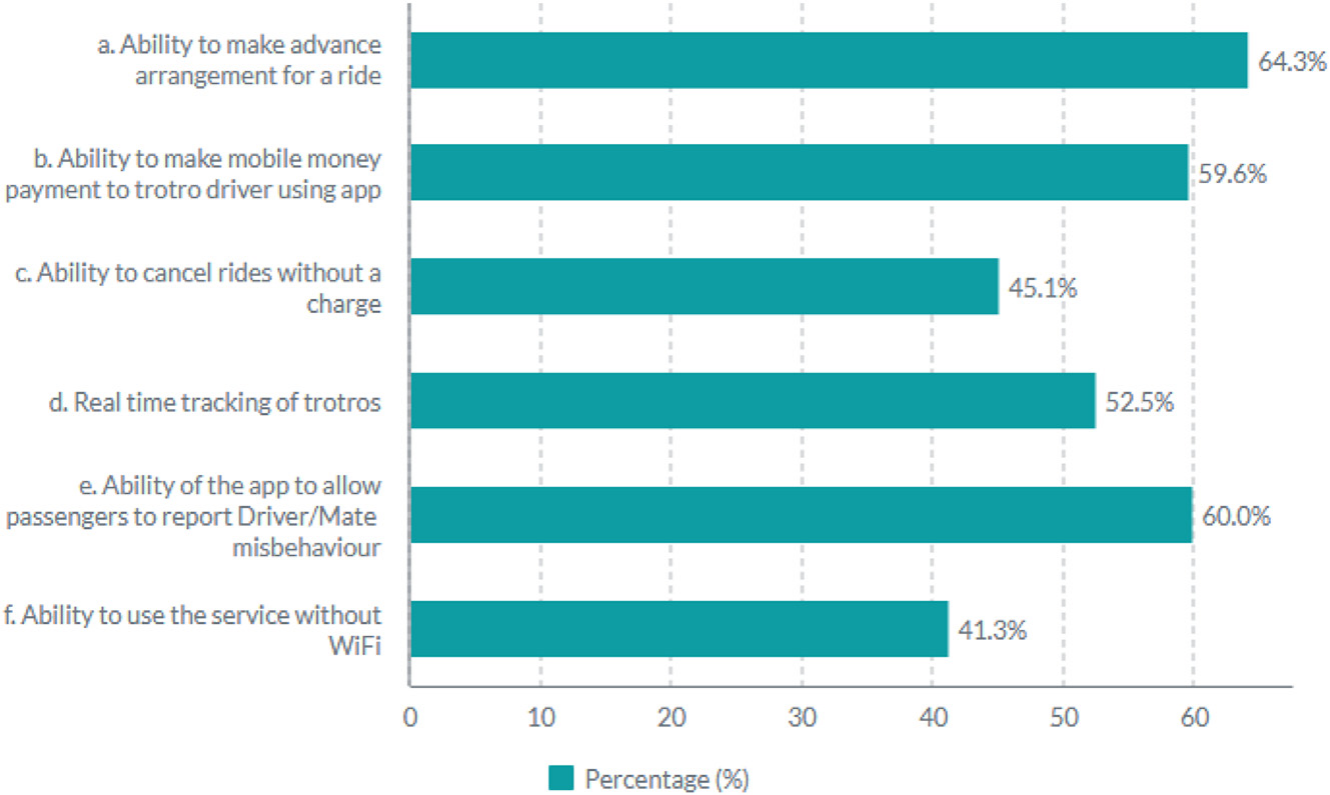
MaaS features for trotro

Assessment of the technologies that were seen as possibly benefitial to the trotro, showed that the most in-demand features (>50%) for the *trotro* service were (i) a booking feature (64.3%), (ii) a driver/mate reporting feature (60%), (iii) a mobile money payment feature (59.6%), and (iv) a real-time tracking feature (52.5%). Figure 1 shows the technology-based solutions that commuters thought could improve their service experiences.

## 4 Discussion

Successfully improving service quality of *trotros* in Ghana could result in the improvement of transport quality for the vast majority of people. Improving service quality however requires understanding the issues most fundamental to commuters in their choice of public transport. This study contributes to the growing literature on *trotro* service quality by retooling the SERVPERF to allow it better capture the construct of variability in the trotro service. The study also assessed the differences in service quality perceptions between able-bodied male respondents and more transport vulnerable groups, and as well, the attractiveness of some technology-based solutions. From the service quality evaluation, the following factors were determined as being critical to commuters' choice of *trotros*: ***reliability***, **v*ariability in cost*** and ***responsiveness***. Among the service quality constructs, reliability was the first most significant factor extracted. The factor contributed 24.9% of the total variance, and was comprised of the variables; ‘*Times between departures of* *Trotros* *are relatively short’*, ‘*Passenger belongings are secure*’, ‘*It is easy to get a space on a* *trotro* *in the morning’*, ‘*It is easy to get a space on a* *trotro* *in the evening’*, ‘*It is safe to use* *trotro* *in the morning*’, ‘*It is safe to use* *trotro* *in the evening*’ *‘I have confidence in the* *trotro* *service operators to do their work’*, ‘*Trotro* *drivers are polite*’, ‘*Trotro* *mates are polite*’, *‘The insides of* *trotro* *buses are generally clean and hygienic*’ and ‘*Trotro* *mates are neat*’. As such, the construct of service reliability, which entails the ability of the operators to provide the promised service dependably and accurately, was considered as a significant factor that impacted *trotro* users. In an earlier study conducted by Sam et al. (2018), the construct ‘reliability’ was also a statistically significant factor that influenced commuters use of *trotros* within the Kumasi metropolis. The findings of this study therefore re-emphasized the importance of this service quality parameter, and corroborated previous research findings (Adarkwa, 1991; Horsu and Yeboah, 2015; Sam et al., 2018) on its importance to commuters. Variability in cost of the service was the second most important factor extracted from the factor analysis. The factor accounted for 14.63% of the total variance. The variables ‘*Trotro* *fares are sometimes higher for women, the old or disabled*’, ‘*Trotro* *fares are sometimes lower for women, the old or disabled*’, *‘Women, the old and disabled face more discrimination in using* *trotros*’ and ‘*Trotro* *fares are sometimes higher for the same route during different times of day*’ loaded unto this factor. In an earlier study, Birago et al. (2017) suggest the cost of public transport did have a significant impact on mode choice in Ghana. As such, the constantly varying fares (Buckman, 2013) of *trotros* could be a factor commuters perceived as affecting service quality. Abenaab (2012) suggests that, the variation in fares can sometimes be due to trotro mates who in some instances, consciously cheat commuters by holding on to change or by overcharging for trips. Persons living with disabilities also noted that, mates charged them twice the normal fares for bringing on board, wheelchairs and other equipment. The results suggest the indiscriminate change in fares were a key service quality concern of commuters. Responsiveness of operators followed next, as the third most important concern of commuters. The factor accounted for 11.37% of the total variance. The variables ‘*Trotro mates tend to be more helpful to customers with disabilities, old people or women with children*’, ‘*Trotro drivers generally tend to respond to passenger concerns*’, ‘*Trotro fares are commensurate with service provided’* and ‘*Fares charged for luggage are affordable*’ also loaded unto this factor. The finding suggests that the willingness of operators to attend to the needs of commuters was another key measure by which commuters measured service quality of *trotros*. The emergence of the two constructs ‘reliability’ and ‘responsiveness’ that were also identified by Sam et al. (2018) as statistically significant *trotro* service quality factors suggested that the constructs had some influence on Ghanaian commuters. Additionally, the study also identified that male and female respondents had differing perspectives of certain service quality constructs. Statistically significant differences (**p≤0.05**) were observed with regard to the perception of *trotro* driver and mates' politeness, *trotro* mates' neatness, the variations in *trotro* fares for women, the perception of discrimination towards women, and the willingness of the respondent groups to use *trotros* if there were other equally affordable modes of transport. Female respondents and persons living with disabilities were more likely to perceive service quality as being poorer than male respondents across these service quality parameters. These findings suggest there could perhaps be differences in the service quality experienced by males and females in their use of *trotros*. This further re-iterated the importance of the construct ‘Variability’ as a service quality measure for paratransit since the informal nature of operations implies the service quality that commuters get could largely depend on the specific operator whose vehicle the individual finds themselves in, and service quality the operator subjectively chooses to offer. The technological features that were considered most in-demand based on the assessment of suitable technologies included: (i) a booking feature, (ii) a driver/mate reporting feature, (iii) a mobile money payment feature, and (iv) a real-time tracking feature. It was also presumed that the selection of these technologies by respondents was because of their abilities in addressing specific challenges. Technologies that could be used in booking, as well as tracking the location of vehicles in real time, could improve *reliability* of the service. Features that could allow commuters report driver/mate misbehavior, could ensure greater *responsiveness* by helping regulators identify bad operators. Also, safe and easy payment options via mobile money (Tobbin and Kuwornu, 2011) could reduce incidences of *varying costs*, and ensure greater accountability on the part of operators. Asked about their intention to continue using *trotro* if service quality challenges were addressed, about 89% of respondents stated they were willing to continue using or increase their use of *trotros* if service quality was improved, and 11 % were of an opposing view.

## 5 Limitations of the study

The most significant limitation of the study was that it used a stated preference approach in examining the technology preference of commuters. This meant they did not actually interact with the technology before stating their intentions, and as such, could have overstated their intention to use these technologies if they were tech-savvy. Less technology enthusiastic individuals could also have understated the value of the technology to them especially if they could not even imagine the technology-based solutions being proposed.

## 6 New findings/Significance of the study

Three key service quality factors: reliability, variability in cost and responsiveness were determined to affect *trotro* users based on the modified SERVPERF used in this study. The study in particular highlights the importance of the construct ‘variability in cost’ to *trotro* users. Existing literature on *trotros* suggest that variation in fares occur for a number of reasons. Firstly, there are legitimate reasons such as service-wide increase in fares (Buckman, 2013), however, for the most part, variations in fares occur when an operator (often the mate) wants to exploit the passenger (Abenaab, 2012; Ofori-Debra, 2017). This study contributes to the literature on *trotros* by highlighting the importance of this construct to perceptions of this mode's service quality. Technologies that seem to help in the improvement of service quality and the closing of service quality gaps in the three identified areas were also seen as appealing features that could be implemented in the *trotro* service. The influence of these technologies on service quality could be studied much closer, and as well, the willingness of all stakeholders to participate in a technology-based *trotro* service. The findings of this study have benefit for transport planners in countries with paratransit, particularly those operated in similar ways to the *trotro*.

## 7 Managerial implications

The findings in this study present new opportunities for transport managers in developing countries to improve the quality of paratransit services. Since transport service provision in developing countries have often been relegated to private actors --with little checks on service quality (Kumar and Barrett, 2008), there is the need to constantly re-examine the ability of these services to deliver quality services to commuters. Technology-based solutions such as the mobility as a service features identified in this study could be explored further as avenues for better regulation of transport services. Technology-based solutions could benefit transport operators, regulators and commuters if retrofitted properly to the local situations in these countries. Technologies that increase the profitability of operators could for instance encourage the participation of operators. Technologies that improve service quality for commuters could as well help address some of the systemic issues with this mode of transport, while helping regulators coordinate transport services. Since paratransit are not exclusive to Ghana, some of the lessons learnt from this study could perhaps be applicable to transport management in other countries with paratransit.

## 8 Conclusion

In this study, the objectives were to modify the SERVPERF tool used in assessing the service quality concerns of *trotros* users, evaluate the differences in service quality between male and female respondent groups, and as well, assess the attractiveness of certain technological features to commuters. From the results, it was determined that, the service quality constructs reliability, variability (in cost) and responsiveness were the three main factors that were of importance to commuters. There were as well, statistically significant differences observed between responses of male and female study participants, and certain technological solutions were determined as possibly beneficial in resolving service quality issues. Technologies that could help commuters make advance arrangements for trips, mobile money payments, track the location of *trotros* and report misbehavior of operators, were considered most in demand. Together, the findings suggest that technologies that can improve reliability of the *trotro*, reduce variability around fares, and increase the accountability of operators could have the most benefit for *trotro* commuters. This suggests there exist service quality gaps that can be filled by MaaS features. However, the willingness of all relevant stakeholders to participate must be examined much more closely to determine the possible success of such a scheme.

## Declarations

### Author contribution statement

Emmanuel Dzisi: Conceived and designed the experiments; Performed the experiments; Analyzed and interpreted the data; Contributed reagents, materials, analysis tools or data; Wrote the paper.

Daniel Obeng Atuah, Yaw Adubofuor Tuffuor: Conceived and designed the experiments; Analyzed and interpreted the data.

### Funding statement

This research did not receive any specific grant from funding agencies in the public, commercial, or not-for-profit sectors.

### Data availability statement

Data will be made available on request.

### Declaration of interests statement

The authors declare no conflict of interest.

### Additional information

No additional information is available for this paper.

## Appendix A Supplementary data

Servqual_Appendix_V2 Servqual_Appendix_V2
