# Supplementary material for: Modifying the SERVPERF to assess paratransit minibus taxis trotro in Ghana and the relevance of mobility-as-a-service features to the service
Source: Heliyon. 2021 May 27;7(5):e07071. doi: 10.1016/j.heliyon.2021.e07071 (PMC8176316; doi:10.1016/j.heliyon.2021.e07071)
Supplement: Servqual_Appendix_V2 [file mmc1.docx]

**Appendix**

**Table 6** Independent Samples Test

| **Independent Samples Test** | | | | | | | | | | |
| --- | --- | --- | --- | --- | --- | --- | --- | --- | --- | --- |
|  | | Levene's Test for Equality of Variances | | t-test for Equality of Means | | | | | | |
|  |  | F | Sig. | t | df | Sig. (2-tailed) | Mean Difference | Std. Error Difference | 95% Confidence Interval of the Difference | |
|  |  |  |  |  |  |  |  |  | Lower | Upper |
| 02 | Equal var assumed | 4.824 | .028 | -1.828 | 908 | .068 | -.18839 | .10309 | -.39071 | .01392 |
|  | Equal var not assumed |  |  | -1.807 | 819.714 | .071 | -.18839 | .10427 | -.39306 | .01627 |
| 03 | Equal var assumed | 4.370 | .037 | -1.110 | 908 | .267 | -.11484 | .10344 | -.31785 | .08817 |
|  | Equal var not assumed |  |  | -1.098 | 821.102 | .273 | -.11484 | .10459 | -.32014 | .09045 |
| 04 | Equal var assumed | 1.323 | .250 | -1.676 | 908 | .094 | -.19062 | .11376 | -.41389 | .03264 |
|  | Equal var not assumed |  |  | -1.666 | 841.122 | .096 | -.19062 | .11444 | -.41525 | .03401 |
| 05 | Equal var assumed | 3.817 | .051 | -.395 | 908 | .693 | -.04167 | .10557 | -.24887 | .16552 |
|  | Equal var not assumed |  |  | -.392 | 834.195 | .695 | -.04167 | .10640 | -.25051 | .16716 |
| 06 | Equal var assumed | 14.048 | .000 | -1.554 | 908 | .120 | -.16536 | .10639 | -.37417 | .04345 |
|  | Equal var not assumed |  |  | -1.534 | 811.879 | .125 | -.16536 | .10782 | -.37699 | .04627 |
| 07 | Equal var assumed | 6.324 | .012 | -.642 | 908 | .521 | -.06623 | .10324 | -.26884 | .13638 |
|  | Equal var not assumed |  |  | -.634 | 818.891 | .526 | -.06623 | .10444 | -.27124 | .13877 |
| 08 | Equal var assumed | 1.481 | .224 | -1.760 | 908 | .079 | -.17802 | .10115 | -.37653 | .02049 |
|  | Equal var not assumed |  |  | -1.746 | 833.458 | .081 | -.17802 | .10196 | -.37815 | .02210 |
| 09 | Equal var assumed | 2.940 | .087 | -3.582 | 908 | ***.000*** | -.33193 | .09268 | -.51383 | -.15004 |
|  | Equal var not assumed |  |  | -3.562 | 843.371 | ***.000*** | -.33193 | .09318 | -.51483 | -.14904 |
| 10 | Equal var assumed | 6.460 | .011 | -3.086 | 908 | ***.002*** | -.28466 | .09224 | -.46569 | -.10363 |
|  | Equal var not assumed |  |  | -3.059 | 829.759 | ***.002*** | -.28466 | .09307 | -.46733 | -.10199 |
| 11 | Equal var assumed | 11.449 | .001 | -.951 | 908 | .342 | -.09048 | .09514 | -.27719 | .09624 |
|  | Equal var not assumed |  |  | -.939 | 814.581 | .348 | -.09048 | .09635 | -.27959 | .09864 |
| 12 | Equal var assumed | .825 | .364 | -3.092 | 908 | ***.002*** | -.28631 | .09260 | -.46806 | -.10457 |
|  | Equal var not assumed |  |  | -3.064 | 829.880 | ***.002*** | -.28631 | .09343 | -.46970 | -.10293 |
| 13 | Equal var assumed | 13.733 | .000 | 1.931 | 908 | .054 | .20284 | .10502 | -.00328 | .40895 |
|  | Equal var not assumed |  |  | 1.906 | 812.049 | .057 | .20284 | .10642 | -.00606 | .41173 |
| 14 | Equal var assumed | 8.146 | .004 | 2.072 | 908 | ***.039*** | .20182 | .09738 | .01069 | .39294 |
|  | Equal var not assumed |  |  | 2.046 | 814.522 | ***.041*** | .20182 | .09862 | .00823 | .39541 |
| 15 | Equal var assumed | 2.784 | .096 | -1.234 | 908 | .217 | -.12273 | .09945 | -.31791 | .07245 |
|  | Equal var not assumed |  |  | -1.226 | 840.230 | .220 | -.12273 | .10007 | -.31915 | .07369 |
| 16 | Equal var assumed | 34.173 | .000 | 3.812 | 908 | ***.000*** | .45422 | .11917 | .22035 | .68809 |
|  | Equal var not assumed |  |  | 3.737 | 782.991 | ***.000*** | .45422 | .12156 | .21560 | .69284 |
| 17 | Equal var assumed | 16.651 | .000 | 2.230 | 908 | ***.026*** | .26869 | .12051 | .03218 | .50520 |
|  | Equal var not assumed |  |  | 2.199 | 809.321 | ***.028*** | .26869 | .12219 | .02883 | .50855 |
| 18 | Equal var assumed | 4.560 | .033 | 2.793 | 908 | ***.005*** | .33741 | .12082 | .10029 | .57452 |
|  | Equal var not assumed |  |  | 2.769 | 831.499 | ***.006*** | .33741 | .12185 | .09824 | .57657 |
| 19 | Equal var assumed | 4.690 | .031 | .534 | 908 | .593 | .05446 | .10194 | -.14561 | .25453 |
|  | Equal var not assumed |  |  | .530 | 835.129 | .596 | .05446 | .10272 | -.14715 | .25607 |
| 20 | Equal var assumed | .008 | .930 | 2.834 | 908 | ***.005*** | .33760 | .11913 | .10380 | .57140 |
|  | Equal var not assumed |  |  | 2.833 | 861.140 | ***.005*** | .33760 | .11917 | .10370 | .57149 |
| 21 | Equal var assumed | 21.603 | .000 | 1.211 | 908 | .226 | .11720 | .09676 | -.07271 | .30711 |
|  | Equal var not assumed |  |  | 1.189 | 787.524 | .235 | .11720 | .09861 | -.07637 | .31076 |
| 22 | Equal var assumed | 7.026 | .008 | -.381 | 908 | .703 | -.04161 | .10917 | -.25586 | .17264 |
|  | Equal var not assumed |  |  | -.377 | 822.846 | .706 | -.04161 | .11033 | -.25818 | .17496 |
| 23 | Equal var assumed | .682 | .409 | 2.953 | 908 | ***.003*** | .34975 | .11842 | .11734 | .58216 |
|  | Equal var not assumed |  |  | 2.957 | 866.155 | ***.003*** | .34975 | .11828 | .11761 | .58189 |
